# The urgent need to adapt working conditions to global warming-driven heat waves to protect workers’ health

**DOI:** 10.47626/1679-4435-2026-1623

**Published:** 2026-10-20

**Authors:** Danielle Campos Bedin, João Silvestre Silva-Junior, Evangelina da Motta Pacheco Alves de Araujo

**Affiliations:** 1 Instituto Ar, São Paulo, SP, Brazil; 2 Faculdade de Medicina da Universidade de São Paulo (FMUSP), Departamento de Medicina Legal, Bioética, Medicina do Trabalho e Medicina Física e Reabilitação, São Paulo, SP, Brazil; 3 Hospital das Clínicas da Faculdade de Medicina da Universidade de São Paulo (HCFMUSP), São Paulo, SP, Brazil

**Keywords:** extreme heat, workers’ health surveillance, occupational health, heat stress disorders

## Abstract

Climate change poses one of the greatest public health challenges of the twenty-first century and directly affects the world of work. Rising global mean temperatures and increasingly intense heat waves are increasing workers’ exposure to heat stress, with substantial consequences for physical and mental health and work capacity. This article discusses scientific evidence on the effects of heat waves on workers’ health, including the underlying physiological mechanisms, clinical consequences, effects on productivity, and economic implications for businesses and supply chains. It also addresses public policies and technical standards governing occupational heat exposure, with particular emphasis on the Brazilian context. Global evidence documents record losses of working hours attributable to heat and mounting economic costs, demonstrating that adaptation to rising temperatures is both a worker health protection measure and a strategy for operational sustainability. Recognizing heat as a structural occupational hazard and incorporating heat-related risks into business management and public policy are therefore essential to protecting workers and ensuring economic resilience in an increasingly warmer climate.

## INTRODUCTION

Climate change is now recognized as one of the greatest threats to global health. The Sixth Assessment Report of the Intergovernmental Panel on Climate Change indicated that the global mean temperature has already risen by approximately 1.1 °C above preindustrial levels, primarily because of anthropogenic greenhouse gas emissions [^1^]. This warming has caused substantial changes in the climate system, including increases in the frequency, duration, and intensity of extreme weather events, particularly heat waves [^2^].

Epidemiologic studies have shown that exposure to high temperatures is associated with increased mortality and hospital admissions as well as the exacerbation of cardiovascular, respiratory, and kidney diseases [^3^-^5^]. Extreme heat has therefore emerged as a major environmental risk factor for human health.

In addition to its direct health effects, rising temperatures have major implications for the world of work. The Lancet Countdown on Health and Climate Change highlights that global warming poses a growing threat to workforce productivity and global economic stability [^6^,^7^]. Aggregated data for 2024 indicate that approximately 639 billion working hours were lost worldwide because of heat exposure, resulting in economic losses exceeding US$1 trillion [^6^].

Outdoor workers in sectors such as agriculture, construction, mining, and transportation are among those most vulnerable to heat exposure, particularly in tropical and subtropical regions [^8^]. Accordingly, understanding the effects of heat waves on worker health has become essential to informing public policy and developing business strategies for climate change adaptation.

### HEAT WAVES AND OCCUPATIONAL EXPOSURE

Heat waves are defined as prolonged periods of abnormally high temperatures relative to the local climate. When accompanied by high relative humidity and limited air movement, these conditions further impair the human body’s ability to dissipate heat [^1^].

In occupational settings, the heat load experienced by workers results from the interaction of several environmental factors, including air temperature, relative humidity, solar radiation, and wind speed [^9^]. These factors affect the body’s ability to dissipate heat and maintain core body temperature within physiologically safe limits.

Several environmental indices have been developed to assess occupational heat risk. The most widely used is the Wet Bulb Globe Temperature (WBGT) index, which integrates the effects of temperature, humidity, radiant heat, and air movement to estimate the heat load experienced by workers [^10^]. The WBGT is widely incorporated into international occupational hygiene standards and serves as an important reference for establishing exposure limits and determining appropriate work–rest schedules for activities performed in hot environments [^10^].

### PHYSIOLOGY OF HEAT STRESS

The maintenance of body temperature is a complex physiologic process regulated by the central nervous system. Under normal conditions, core body temperature remains close to 37 °C because thermoregulatory mechanisms maintain a balance between heat production and heat dissipation [^11^].

When the body is exposed to a hot environment or intense physical exertion, metabolic heat production increases. To prevent excessive heat accumulation, the body activates compensatory mechanisms, primarily sweating and peripheral vasodilation. Sweating promotes heat loss through evaporation, whereas peripheral vasodilation increases heat transfer to the skin surface [^12^]. When the environmental heat load exceeds the compensatory capacity of these mechanisms, heat strain develops. The body then exhibits progressive signs of physiologic stress, including an elevated heart rate, dehydration, reduced plasma volume, and increased cardiovascular strain [^13^].

If heat exposure persists, more severe clinical manifestations may develop, including heat exhaustion and heat stroke. Heat stroke is characterized by severe hyperthermia accompanied by neurologic dysfunction and can rapidly progress to multiorgan failure and death without immediate treatment [^14^].

Recent evidence also indicates that repeated heat exposure may have cumulative adverse effects on the kidneys, resulting in acute kidney injury and chronic kidney disease associated with occupational heat exposure [^15^,^16^].

### EFFECTS ON PRODUCTIVITY AND OCCUPATIONAL SAFETY

Extreme heat has substantial effects on workers’ physical and cognitive performance. During physical exertion in hot environments, the body redirects a portion of the blood flow to the skin to facilitate heat dissipation. This redistribution of blood flow, together with cardiovascular strain and dehydration, contributes to earlier fatigue and reduced work capacity [^8^].

Occupational heat exposure is associated with significant reductions in productivity, particularly in jobs involving moderate or high physical demands. Systematic reviews have shown that increasing heat stress reduces work capacity, increases the need for rest breaks, and impairs physical performance. Field studies have estimated an average productivity loss of approximately 2.6% for every 1 °C increase in WBGT above 24 °C, although the magnitude of this effect varies according to the activity performed, workers’ acclimatization status, and the mitigation strategies adopted [^8^,^17^].

In addition to its physical effects, extreme heat impairs essential cognitive functions, including attention, working memory, and decision-making. These impairments increase the risk of workplace accidents and operational errors, particularly during activities involving vehicle or machinery operation [^18^,^19^].

Climate models indicate that, by the middle of the twenty-first century, global warming could substantially reduce work capacity in tropical regions, with major consequences for agricultural and industrial productivity [^20^,^21^].

### ECONOMIC IMPACTS

Extreme heat also has major economic consequences. In addition to reducing productivity, heat exposure is associated with increased absenteeism, occupational fatigue, and workplace accident rates, as well as higher costs related to medical care, social security-related work leave, and workforce turnover [^8^,^17^,^19^]. Evidence shows that heat stress progressively reduces workers’ physical and cognitive capacity, resulting in a slower work pace, a greater need for recovery breaks, and a higher likelihood of operational errors, particularly in activities requiring intense physical exertion or rapid decision-making [^17^,^18^].

From a microeconomic perspective, the effects of heat extend beyond the direct loss of productivity among individual workers. Reduced performance at critical stages of production can disrupt schedules, increase operating costs, reduce logistical efficiency, delay the delivery of materials and products, and undermine business competitiveness [^20^,^21^]. In highly integrated production systems, these losses tend to be amplified because localized disruptions affect suppliers, distributors, and end consumers. Recent studies have shown that the effects of extreme heat events can propagate throughout global supply chains, producing economic consequences far greater than those observed only in the regions directly affected by the climatic event [^22^,^23^].

One issue that remains insufficiently addressed in Brazilian occupational regulations is that heat risk assessment continues to focus predominantly on protecting individual workers, even though the effects of heat occur across multiple organizational and economic levels. Although Regulatory Standard No. 15 (NR-15) and Occupational Hygiene Standard No. 06 (NHO-06) are essential instruments for characterizing and assessing occupational heat exposure, both focus primarily on individual exposure and place less emphasis on the operational and economic consequences of reduced collective work capacity. In practice, diminished performance by a single worker can disrupt entire teams, reduce production-line efficiency, delay logistics operations, and increase costs at multiple stages of the supply chain. Heat adaptation should therefore be understood not only as an occupational health protection measure but also as a strategy for managing productivity, operational continuity, and economic resilience in the face of climate change [^6^,^21^-^26^].

Heat risk should thus be understood not only as an occupational health concern but also as a determinant of the economic resilience of organizations and production systems. As climate change drives increases in the frequency, duration, and intensity of heat waves [^1^,^2^,^6^], Brazilian public policies and regulatory standards must be strengthened by incorporating preventive approaches, continuous monitoring of environmental conditions, real-time meteorological data, and adaptation strategies tailored to outdoor work [^24^-^26^]. Investments in prevention, planning, and adaptation have the potential to reduce both adverse effects on worker health and the economic losses resulting from declining productivity and the increased vulnerability of supply chains [^6^,^21^-^23^].

### PUBLIC POLICIES AND THE BRAZILIAN CONTEXT

Brazil has an important regulatory framework for managing occupational heat exposure. Legal protections for workers exposed to heat are based primarily on Annex 3 of NR-15 and NHO-06, issued by the Fundação Jorge Duprat Figueiredo de Segurança e Medicina do Trabalho. NR-15 establishes the legal criteria for determining whether heat exposure constitutes an unhealthy working condition, whereas NHO-06 defines the technical methodology for assessing occupational heat stress using the WBGT index. This assessment considers environmental variables, the metabolic rate associated with the activity performed, worker acclimatization, and the clothing worn [^24^,^25^].

Despite the methodological rigor of NHO-06, its application to outdoor work has limitations. The WBGT is directly influenced by highly dynamic environmental factors, including the intensity of solar radiation, cloud cover, wind speed, and relative humidity, all of which may vary substantially over the course of a single work shift [^25^].

In tropical countries such as Brazil, where many workers routinely perform their duties in direct sunlight, these fluctuations make it difficult to obtain a single measurement that adequately represents the heat load experienced throughout the entire work shift. Moreover, the assessment requires properly calibrated specialized equipment and trained professionals, making its routine use in labor inspections operationally complex, particularly for outdoor activities conducted across extensive geographic areas.

Another frequently discussed issue is the persistent limitation of Brazilian legislation in adequately addressing exposure to natural heat. Historically, Annex 3 of NR-15 restricted the recognition of unhealthy working conditions primarily to exposure arising from artificial heat sources, leaving workers exposed exclusively to solar radiation with less legal protection [^24^]. The Regulatory Impact Analysis Report prepared by the Brazilian Ministry of Labor and Employment acknowledges that this exclusion stems more from the methodological challenges involved in assessing outdoor environments than from an absence of occupational risk. The report also emphasizes the need to develop specific criteria for workers exposed to solar heat and to incorporate complementary environmental monitoring tools [^26^].

These limitations have become increasingly consequential in the context of climate change, which has increased the frequency, intensity, and duration of heat waves throughout Brazil [^1^]. Standards developed based on historical climatic conditions may no longer adequately reflect current occupational exposure patterns, particularly in tropical regions, where workers frequently operate near the physiological limits of heat tolerance for much of the year. In this context, we advocate strengthening Brazilian regulations by incorporating continuous monitoring strategies, real-time meteorological data, periodic updates to exposure limits, and specific protocols for outdoor work, thereby improving the prevention of heat-related illnesses and expanding protections for worker health [^26^].

## CONCLUSIONS

Climate change is exacerbating workers’ exposure to high temperatures, requiring the mitigation of this occupational hazard and adaptation to new climatic conditions to be treated as global priorities. Heat waves directly affect worker health, impair physical and cognitive performance, and generate substantial economic consequences for businesses, production systems, and health and social security systems.

Recognizing heat as a structural occupational hazard is essential for guiding public policy, strengthening labor regulations, and promoting climate adaptation strategies across business operations. Protecting workers as global temperatures rise is not only a public health concern but also an economic and social imperative in an increasingly hot world.

## Data Availability

The data supporting the findings of this study are available within the article.
